# Nanotreatment and Nanodiagnosis of Prostate Cancer: Recent Updates

**DOI:** 10.3390/nano10091696

**Published:** 2020-08-28

**Authors:** Mahmood Barani, Fakhara Sabir, Abbas Rahdar, Rabia Arshad, George Z. Kyzas

**Affiliations:** 1 Department of Chemistry, Shahid Bahonar University of Kerman, Kerman 7616914111, Iran; dan.mahmoodbarani@sci.uk.ac.ir; 2 Institute of Pharmaceutical Technology and Regulatory Affairs, Faculty of Pharmacy, University of Szeged, Eötvös u. 6, H-6720 Szeged, Hungary; sabir.fakhara@pharm.u-szeged.hu; 3 Department of Physics, Faculty of Science, University of Zabol, Zabol 538-98615, Iran; 4 Department of Pharmacy, Quaid-i-Azam University, Islamabad 45320, Pakistan; rabia.arshad@bs.qau.edu.pk; 5 Department of Chemistry, International Hellenic University, 65404 Kavala, Greece

**Keywords:** nanomaterials, cancer, prostate, treatment, diagnosis

## Abstract

The fabrication and development of nanomaterials for the treatment of prostate cancer have gained significant appraisal in recent years. Advancements in synthesis of organic and inorganic nanomaterials with charge, particle size, specified geometry, ligand attachment etc have resulted in greater biocompatibility and active targeting at cancer site. Despite all of the advances made over the years in discovering drugs, methods, and new biomarkers for cancer of the prostate (PCa), PCa remains one of the most troubling cancers among people. Early on, effective diagnosis is an essential part of treating prostate cancer. Prostate-specific antigen (PSA) or serum prostate-specific antigen is the best serum marker widely accessible for diagnosis of PCa. Numerous efforts have been made over the past decade to design new biosensor-based strategies for biomolecules detection and PSA miniaturization biomarkers. The growing nanotechnology is expected to have a significant effect in the immediate future on scientific research and healthcare. Nanotechnology is thus predicted to find a way to solve one of the most and long-standing problem, “early cancer detection”. For early diagnosis of PCa biomarkers, different nanoparticles with different approaches have been used. In this review, we provide a brief description of the latest achievements and advances in the use of nanoparticles for PCa biomarker diagnosis.

## 1. Introduction

Prostate cancer is one of the major causes of morbidity and mortality in developing and under-developed countries [1]. The most frequent non-skin cancer causing second largest number of deaths in men as compared to other cancers [2]. Prostate cancer can be localized and advanced depending upon its severity [3]. Prostate cancer can metastasize via the lymphatic system and invade into bones [4]. Various factors like age, genetics, environmental toxins, chemical hazards and radiations seem to be involved in the pathogenesis of prostate cancer but the exact mechanism is still unknown [5]. Androgens are involved in the normal developmental phase of prostate and their functions, but, in that phase, they can still steep towards carcinogenesis [6]. Similarly, hyperinsulinaemia accompanying insulin resistance and obesity can directly surge the prostate cancer risk [7]. Various treatment protocols are being practiced to reduce the above-mentioned risk of prostate cancer, and no treatment is required for benign stage cancer [8]. Moreover, in the case of metastatic invasion, surgery can be opted to remove prostate glands and associated tissues and lymph nodes [9]. Radiation therapy is also common in the treatment of prostate cancer [10]. Prostate cancer radiation therapy can be carried in two ways, i.e., external beam radiations and internal radiations (brachytherapy) [11]. Radiations inside the body involve the placement of small radioactive seeds to deliver a very low optimized dose of radiation for relatively long duration via ultrasound imaging guided needle to be supervised by a physician [12,13]. The third most important treatment protocol towards prostate cancer is hormone replacement therapy [14]. Hormone therapy is practiced to hamper the production of male sex hormone (testosterone) [15]. Diminished testosterone level supply is associated with the slow progression of cancer cells [16]. Therefore, luteinizing hormone releasing agonists (leuprolide, goserelin, triptorelin) are preferred to antagonize testosterone levels via preventing the testicles from synthesizing it [17]. Some anti-androgenic medications (bicalutamide, flutamide) are required to prevent the testosterone from reaching cancerous cells [18]. In severe cases, orchiectomy can be performed for removing testicles and diminishing testosterone levels [19]. Freezing of prostate tissues is also being practiced for killing cancer cells [20]. In case of non-responding effects of hormone replacement therapy, chemotherapy can be preferred via using chemotherapeutic agents, i.e., docetaxel and paclitaxel to kill devastating and highly invasive cancer cells [21]. Unfortunately, all these treatment protocols are envisioned with erectile dysfunction, libido, obesity, and bone mass loss [22]. Several diagnosis techniques for prostate cancer have been developed i.e., physical examination, magnetic resonance imaging (MRI), prostate-specific antigen (PSA) testing, biopsy, and staging [23]. Diagnosis of prostate cancer is challenging owing to the vast existence of gaps accompanying over-testing, over-diagnosis, over-treatment, and the non-specificity and heterogenous nature of prostate cancer [24].

Nanomedicine has revolutionized the field of medicine and diagnosis to bypass conventional treatment protocols in the treatment of notorious cancers and various intracellular diseases [25,26]. Enhanced transmembrane penetration, enhanced permeability retention, increased solubility, and targeted drug delivery can be also achieved with the application of nanotechnology to medicine [27,28,29]. Nanoparticles can accumulate in the tumor tissues via active and passive targeting [30]. Active tumor targeting involves the selection of specified ligand receptor which is over expressed in tumor cells. Specified ligand can be anchored with the nanocarriers and they can bind to the over-expressed site of cancer cells for targeted drug delivery [31]. Some common examples of ligands for tumors are folate, transferrin, and galactosamine [32]. Passive targeting can be achieved via the permeation of drug loaded nanocarrier into the leaky vasculature of the tumor [33,34,35,36]. Nanotechnology in prostate cancer also leads to the advanced stealthing [37]. Stealthing results in the increased reaching capability of nanoformulation into tumor site with enhanced circulation time in the blood stream via coating of hydrophilic polymers which results in the induction of strong stealth effects [38]. The mechanistic approach beside stealthing is the evading the path of nanoparticles from mononuclear phagocytic system trap and preventing the early elimination [39]. In reference to prostate cancer, poly(ethylene glycol) (PEG) coated pegylated nanoparticles were accompanied with immense accumulation of nanoparticles at tumor site as compared to un-modified non-stealth nanoparticles [40]. Moreover, other hydrophilic polymers such as dextrans, heparins, and polyvinylpyrrolidone can also be used to induce stealthing effect [41]. Therefore, various types of nanomedicines including liposomes, niosomes, lipid hybrid nanoparticles, polymer–drug conjugates, polymeric nanospheres, nanomicelles, metallic nanoparticles, and immune-conjugates have been successfully synthesized and elevated the quality of life of prostate cancer patients [42,43]. Until now, numerous works have been published investigating the use of nanomaterials to environmental applications [44,45,46,47,48,49]. In contrast, the most pressing challenge is application of nanotechnology to design of multifunctional, structured materials able to target specific diseases and protection of therapeutic moieties [50,51,52,53,54,55,56,57,58,59]. Functionalities to allow transport across biological barriers and to realize the desired clinical benefits rapidly via understanding of toxicological implications of nanomedicines relate to the specific nanoscale properties [60,61,62,63,64,65,66,67,68,69]. The potential environmental impact and a safety assessment of all manufacturing processes require a case-by-case approach to clinical and regulatory evaluation of each nanopharmaceutical.

Nanotechnology has been immensely involved in the detection of prostate cancer biomarkers with marked sensitivity as compared to the conventional enzyme-linked immunosorbent assay (ELISA) method [70]. Higher sensitivity of the nanoparticle anchored detectors is associated with cost-effectiveness because only traces of biomarkers are required for the detection of prostate cancer [71]. Moreover, higher sensitivity also facilitates the screening of prostate cancer via urine instead of blood sample [71]. Blood serum sampling requires high professionalism and is linked with patient non-compliance [72]. Specified nanotechnology-based detectors can efficiently minimize the overdiagnosis and underdiagnosis of cancer due to their excellent specificity and sensitivity, respectively, and could monitor the disease for people at risk of recurrence after recovery [73]. The conventional method needs to be carried out by a professional, and thus, it cannot be a point-of-care for the general population to use [74]. Therefore, magnetic nanoparticles (MNPs) served as the precursor contrast agents for most novel MRI and CT diagnostic technologies for prostate cancer. MNPs can be either used in the form of superparamagnetic iron oxides (SPIOS) and ultra-small SPIOs (USPIOS). Various successful clinical trials have been made with resulting enhanced biocompatibility and reduced toxicity. Iron-oxide based MNPs have been approved as MRI contrast agents by US FDA. Moreover, cationic lipid nanoparticles were also experimented to be attached with SPIOs to overcome low efficacy [74]. Novel photoacoustic imaging (PAI) is an emerging non-invasive imaging technology for prostate cancer which usually combines the laser light and ultrasound effects. Some metallic nanoparticles, i.e., gold nanoparticles can be a wonder source of PAI, based on the mechanistic approach of surface plasmon resonance to enhance the absorption. Similarly, the detection of prostate cancer via nanotechnology at a molecular level can be accompanied through prostate-specific antigen (PSA) as they are potential nanoparticle targets [75].

## 2. Nanomaterials: Applications in Treatment of Prostate Cancer

**Figure 1:**
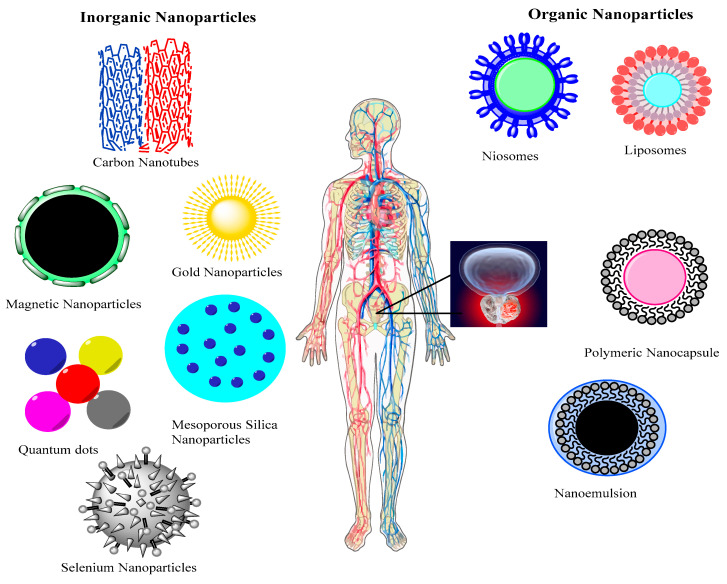
Applications of inorganic and organic nanomaterials in treatment of prostate cancer. Reproduced from [79] with permission from DeGruyter, 2017.

Nanomaterials have been in use for a number of applications, especially for treatment and targeting diseases in the last decade. A carrier system must be biocompatible, inert and can carry a high concentration of drug efficiently. According to present knowledge, most of carrier systems are not able to deliver drug at high concentrations due to their increase cytotoxicity at targeting site. Therefore, many present treatment strategies cannot be used for treatment of cancers specifically breast tumor in females and prostate cancer in males [76]. Prostate cancer (PCa) is one of the most common diseases and its targeting needs greater concentration of active to the organ and tissues affected by malignancy. Both organic (nanoemulsions, liposomes, niosomes, and polymeric nanocapsule) and inorganic (carbon nanotubes, gold nanoparticles, magnetic nanoparticles, silica mesoporous nanoparticles, quantum dots, selenium nanoparticles) are types of nanocarrier that have shown greater efficacy as drug delivery systems for greater number of active pharmaceutical agent (API) for targeting prostate tumor as shown in Figure 1 [77]. The following nanomaterials are capable of increasing active (receptor mediated endocytosis and decorating of nanoparticles with different ligands helps to achieve active targeting) targeting and passive targeting (enhanced permeability and retention effect EPR) [78].

### 2.1. Mesoporous Silica Nanoparticles

MSNs (mesoporous silica nanoparticles) have been used broadly for many research purposes as it is comprised of cationic quaternary ammonium surfactants. MSNs possess properties, including uniform structures, large surface area, modified pore size and have been used in adsorption, immobilization of enzymes. These particles also have property to be used as sensing materials for electrochemical sensors. Surface ligated Ga-Au encapsulated mesoporous silica are one of the important nanomaterial for treatment and diagnosis of prostate cancer [80]. Chuanlam Gu and coworkers used Ga-Au loaded mesoporous silica nanoparticles for the photothermal treatment of the prostate cell lines. The evaluation of these nanoparticles was performed on cancer cell lines (LNCap and DU145). In vitro evaluation data established that Ga-Au@mSiO_2_ efficiently showed the photothermal treatment can abolished the prostate cancer cells [81]. And it was interesting to note that GaAu@mSiO_2_ + NIR photothermal therapy destroys the prostate cancer cells. The present data, showed that surface ligand Ga-Au encapsulated mesoporous silica nanoparticles inhibit the growth of prostate cancer cells and show significant anti-tumor effect in vitro cell line study. Therefore the following study suggests that Ga-Au@mSiO_2_ + NIR particles can be used as promising approach to target the cancer therapy [82].

Huan wang and coworkers used mesoporous silica nanoparticles due to high selectivity, sensitivity and as electrochemical immunosensors to detect and assist treatment of tumor. PSA (prostate-specific antigen) is the most validated tool for prostate malignancy. These researcher developed label free electrochemical immunosensor for prostate specific antigen based on silver hybridized mesoporous silica nanoparticles for targeting prostate tumor [83].

Badr et al. prepared silica nanoparticles loaded with venom obtained from *Walterinnesia aegyptia* (WEV). The following study measured and compared the impact of WEV on apoptosis, proliferation, invasion, and migration of prostate tumor cells either alone or with silica nanoparticles. The nanoparticles decreased the viability of all cell tested (PC3, PCa, LNCaP, isolated from samples of patients) as measured by MTT assay. The IC_50_ values were determined to be 10 and 5 μg/mL for WEV alone and WEV + NP, respectively. WEV + NP decreased the surface expression of the chemokine receptors (CKRs) CXCR3, CXCR4, CXCR5 and CXCR6 to a greater extent than WEV alone and subsequently reduced migration and the invasion response of the cells to the cognate ligands of the CKRs (CXCL10, CXCL12, CXCL13 and CXCL16, respectively). It is also proved that silica nanoparticles modified the cell cycle of PCa and enhanced the apoptosis of the cell. Silica NPs also modified the charge of membrane in mitochondria in the PCa cells and it is also evaluated that sustained delivery of nanoparticles carrying WEV venom is an efficient treatment for prostate tumor [84].

### 2.2. Selenium, Magnetic and Gold Nanoparticles

Bioderived silver (Ag) and gold (Au) nanoparticles have been offering new ways for the treatment of prostate tumor. AuNPs and AgNPs have significantly increased the functionalization that make them candidate agents for conventional chemotherapeutic. Biosynthesized AuNPs and AgNPs showed evidence of having anticancer effects against prostate tumor cell lines, but further studies can help to evaluate the biocompatibility and safety profile of the following NPs in other body tissues. The in vivo models NPs do not show any information that is relevant to toxicity of these NPs, although these researches are required to support biogenic AuNPs or AgNPs retention, clearance, uptake, pharmacodynamics, and pharmacokinetics.

Hu and coworkers developed nanoparticles with surface attached glucose for specific uptake into rapidly dividing cells. In following study researcher developed an array of functionalized gold nanoparticles and modified their attributes for different applications. The modification in functional property of biomolecule on the GNPs can change the biological activities in cancer cells. Glucose-coated GNPs (Glu-GNPs) are produced based on cancer cell metabolism, and can be selectively taken up by malignant cells and directed in the cell cytoplasm. The study also evaluate that how glucose can increase the cellular uptake of GNPs and how GNPs can alter the radiation cytotoxicity in prostate cancer [85,86].

Zhang and coworkers developed and evaluated thio glucose coated/capped GNP (gold nanoparticles) to inhibit growth and to enhance radiation sensitivity in prostate cancer cells. Human prostatic cell carcinoma cell line DU-145 was screened and irradiation was measured using a standard colorimetric MTT assay. Results of Glu-GNPs nanoparticles showed enhanced radiation and toxicity in prostate cancer cells [87].

Rastinehad et al. used nanoparticles made from Au-silica for ultrafocal photothermal ablation of prostate cancer. Au-silica NPs could absorb near-infrared light at high tissue transparency wavelengths and provide a highly localized light-based strategy for the treatment of prostate cancer with low side effects [88].

Ravi Shukla et al., worked on prostate tumor specific epigallocatechin-gallate (radioactive gold nanoparticles). These researcher work on hypothesis that radioactive nanoparticles when delivered intratumorally will circumvent the transport pathway, that will result in therapeutic delivery. The developed gold nanoparticles have AU-198 isotope, the range of 198Au up to 1100 cell diameters or 11mm in tissues is longer to provide radiation dose to cells in prostate glands and shorter to lessen the radiation dose to tissues close to the periphery. The following biocompatible formulation of 198AuNPs as modified gold salt into gold nanoparticles and selectively bind with excellent affinity to Laminin67R receptors that are overexpressed in PCa. Therapeutic and pharmacokinetic studies showed more then 80% reduction of tumor volume showing prominent inhibition of tumor development as compared to control group. The following study showed novel 198AuNP-EGCg therapeutics may provide prominent progress in treatment of prostate tumor [89].

**Figure 2:**
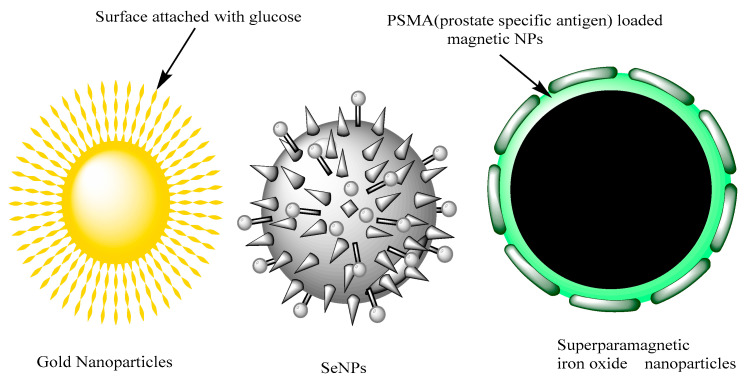
Glucose capped gold nanoaprticles and SeNPs and Superparamagnetic iron oxide nanoparticles for treatment of PCa. Reproduced from [79] with permission from DeGruyter, 2017.

Nanoscale selenium (Se) has a broad spectrum of biomedical applications. SeNPs have prominent effect in reduction of oxidative stress; these nanoparticles have remarkable effect as an anticancer agent. The advantage of SeNPs is the zero oxidation state which represents significant increasing bioavailability and quite low toxicity compared to other oxidations states [90]. Anti-prostatic effects of SeNPs are govern by its ability to inhibit the growth of highly proliferating cells via induction of cell cycle arrest at cell division [91]. The targeting of cancer through SeNPs will modified the biomechanical properties of malignant cells and reduce the adhesion force. Besides the direct and potential anticancer effects of SeNPs the small size of these nanoparticles permit more efficient and selective cellular uptake by cell types and specific drug accumulation at target sites as shown in Figure 2 [92,93].

Another, more commonly, used nanomaterial against PCa treatment is magnetic nanoparticles that efficiently produce the heat upon electromagnetic stimulation after preferred accumulation into PCa sites. Besides these, Yu et al. thermally cross-linked superparamagnetic iron oxide nanoparticles to treat prostate cancer. These agents are used as theranostics and are capable of prostate cancer diagnosis via MRI (magnetic resonance imaging) and specific delivery of therapeutics at cancer site [94]. They developed PSMAs (prostate specific membrane antigens) that are capable of binding towards prostate cancer cells in all in vivo and in vitro studies when analyzed via MRI. The results of study showed that the PSMA doxorubicin conjugate has potential for use in novel prostate cancer specific nanotheranostics [95].

### 2.3. Quantum Dots

Quantum dots (QD) are applied to targeted delivery in PCa. Their size is in the range of 2–100 nm with modified optical properties. QD have crystalline metalloid structure and quantum limiting effect at very small size. In vivo studies suggested that QD probes can be delivered to malignant cells by both active and passive targeting (via enhanced permeability and retention effect). For active target delivery, antibody ligated QDs used to target PSMA. PSMA was selected as one of the key-target for both therapeutic and diagnostic purpose in PCa treatment. The retention and accumulation of antibody PSMA at the site of malignant cells is the basis of targeting and scanning for PCa [96,97].

### 2.4. Carbon Nanotube

Carbon nanotubes (CNTs) are used as new site-oriented compound for targeting prostate cancer (targeted drug delivery). CNTs represent chemical, mechanical and physical attributes which make them efficiently biocompatible carrier to deliver anti-neoplastic agents to target prostate tumor. The hexagonal configuration of carbon atoms demonstrates the potential of CNTs for the site-specific delivery of active agents, including proteins, nucleic acid, and other low molecular weight compounds. The principle molecule that is linked to PCa (prostate cancer) is prostate cancer antigen type 3 (PCA3). The following prostate cancer specific antigen overexpressed in all types of cancers. Li et al. studied human PCa cell line with respect to carbon nanotubes [98]. The developed novel system includes SiRNa delivery by using CNTs which was bound and functionalized with amine 1,2-distearoyl-sn-glyceo-3-phosphoethanomaine-*N*-(amino (polyethylene glycol) DSPE-PEG 2000 maleimide and poly(ethylenimine) for targeting and further conjugating with NGR (Asn-Gly-Arg) peptide [99]. The following system more significantly crosses the human PCa-3 membrane in vitro and enhances suppression of dividing cells along with severe apoptosis. Another combinatorial therapy of RNAi along with near infrared photothermal enhanced the anti-tumor activity without producing any other toxic effects [100].

### 2.5. Polymeric Nanoparticles with Block Copolymers

**Figure 3:**
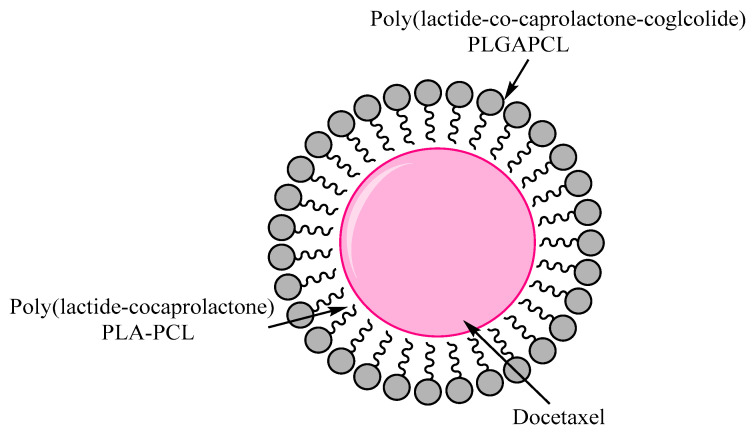
Polymeric PLGA-PCL and PLA-PCL biodegradable nanoparticles loaded with Docetaxel for targeting PCa. Reproduced from [79] with permission from DeGruyter, 2017.

Sanna et al. developed and evaluated the block copolymers (PLGAPCL (poly (lactide-*co*-caprolactone-*co*-glcolide) and PLA-PCL (poly (lactide-*co*-caprolactone)) that are biodegradable and loaded with docetaxel. The cell line study on PCs cells showed higher antiproliferative activity of PLGA-PCL-Dtx NPs compared to free drug. Sawicki et al. investigated the use of polymeric nanoparticles to target a DT-A (diphtheria toxin gene) derived from prostate specific promoter to cells. The injection of DT-A gene investigation has led to prominent decrease in the size of prostate tumor and gland, where direct injection produced zero or less effect. Langer and Farokhzad developed the drug delivery carrier for biocompatible polymeric nanoparticles and aptamers to target PSMA [76,101,102,103]. The potential of these biocompatible polymeric nanoparticles was investigated with in vitro and in vivo studies for uptake and targeted delivery of Dtx by PCa cells. More complicated and complex NPs systems are required to target the PCa as well as many cancer diseases that combine both therapeutic and diagnostic agents as shown in Figure 3 [104].

Dhar et al. developed and used a different strategy to deliver cisplatin to prostate cancer by developing Pt (IV) encapsulated with PSMA (prostate-specific membrane antigen) of PLGA ploy(ethylene glycol)(PEG)-poly(d,l-lactic-*co*-glycolic acid) functionalized controlled polymers. By using PLGA-*b*-PEG nanoparticles with PSMA targeting aptamers (apt) on the surface as a carrier for the platinum compound, a significantly lethal dose of cisplatin was targeted particularly to PCa cells. The results showed that the efficacy of PSMA targeted Pt-NP-Apt nanoparticles for the PSMA is approximately greater than that of free cisplatin [105].

### 2.6. Liposomes

**Figure 4:**
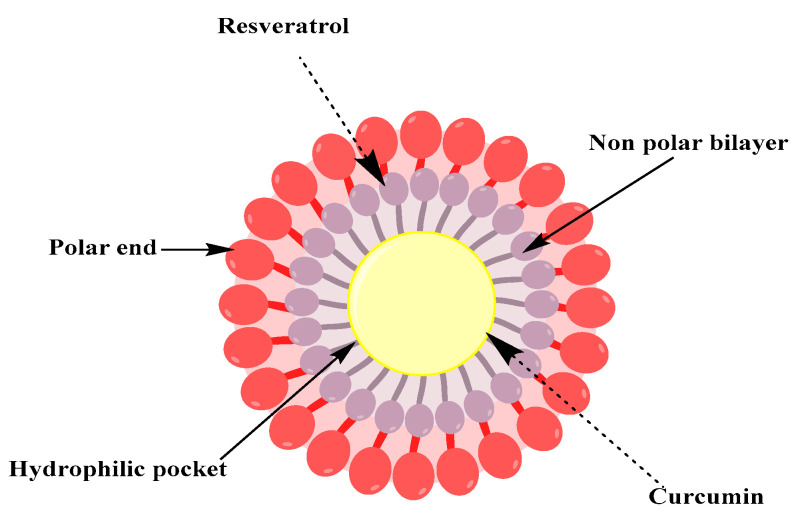
Liposomes co-encapsulated with curcumin and resveratrol for PCa delivery. Reproduced from [79] with permission from DeGruyter, 2017.

Thangapazhem et al. synthesized novel nanoparticles for targeting PCa to deliver curcumin, via loading these molecules into liposomes coated with PSMA specific antibodies. The treatment of human PCa cell lines with curcumin liposomes demonstrated dramatic inhibition of cell division without having any effect on cell viability as shown in Figure 4 [106,107].

Narayanan et al. used resveratrol along with curcumin against PCa treatment and as preventive agent. In this study, liposomes encapsulated with resveratrol and curcumin in male B6C3F1 and PTEN mice. In vitro assays used PEN-CAP8 cancer cells were performed to evaluate the combined effects of curcumin with resveratrol on activated p-Akt cyclin D1, cell growth, cell cycle and apoptosis and androgen receptor proteins involved in tumor proliferation. Analysis with HPLC and prostate tissues showed a prominent increase in curcumin concentration, when liposome was loaded with curcumin co-encapsulated with resveratrol that decreased the prostatic adenocarcinoma. Both these phytochemicals efficiently inhibited the cell growth and produced apoptosis. The observations of following study provide the information that phytochemicals in combination to increase the chemo-preventive effect in prostate tumor [108]. This study suggested reduction of prostate tumor due to loss of tumor suppressor gene PTEN.

Banerjee et al. developed liposomes loaded with doxorubicin; these can deliver the active compound to PCa cells that overexpressed sigma receptors. PEG (polyethylene glycol) phospholipids was attached to surface of DOX-loaded liposomes. Transgenic mice or (DU-145) injected with DOX liposomes has led to inhibition of growth with reduce toxicity. The efficacy of free DOX was associated with significant cytotoxicity. This study confirmed the efficient targeting of liposomes to sigma receptors expressing PCa in both in vitro and in vivo cell study [106].

### 2.7. Nanoemulsion

A new approach for co-encapsulating paclitaxel and herceptin to develop a treatment for advanced PCa has been fabricated by different groups [109,110,111]. The HER2 receptors are overexpressed in some PCa cells, the herceptin has been considered as PCa cells targeting agent. A study showed that oil droplets in nanoemulsion, with herceptin molecules attached to surface, are able to target the HER2 overexpressing cells [112]. The formulation with trastuzumab-along with emulsion containing the active paclitaxel palmitate was checked on PCa cells and on transgenic mice (with induced PCa) [113]. There was no allergic reaction observed during the study and the results were better than other reported drug treatments in inhibiting the PCa cell proliferation [114].

### 2.8. Niosomes

Niosome is a bilayer non-ionic surfactant and cholesterol-based system. Akbarzadeh et al. used anti-cancer drugs on prostate cancers cells and formulated doxycycline loaded niosomes as a carrier system. The in vitro and in vivo study against PCa cells (PC3) showed enhanced chemotherapy effects but increasing biocompatibility for normal cell lines [115]. The increasing anticancer effect was related to the genes in cell cycle of PC3 cells after treating with niosomal formulation [116]. These carriers could be served as efficient delivery system for targeting prostate cancer. Anti-cancer effect of niosomes on PC3 (prostate cancer cell lines) was measured via MTT assay, gene expression, and flow cytometry [116].

## 3. Nanomaterials towards Diagnosis and Biosensing of Prostate Cancer

**Figure 5:**
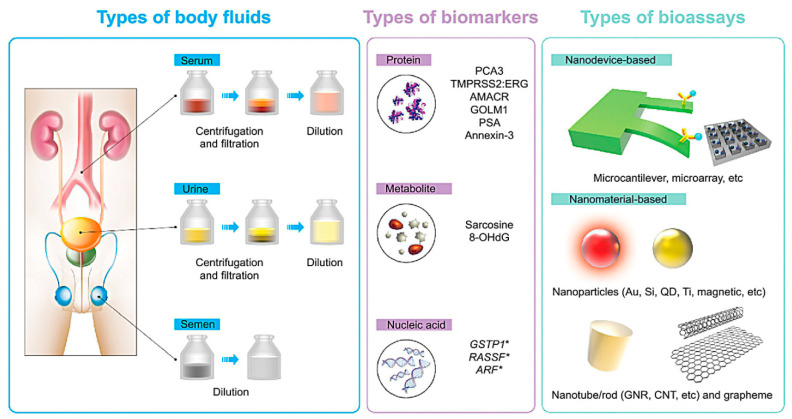
Nanotechnology-based diagnostic approaches (nanobiosensors) involving various biomarkers in various body fluids. Reproduced from [134] with permission from DovePress, 2015.

Prostate cancer is fifth cause of cancer death in the world and the second abundant cancer among men [117,118,119]. Currently, imaging techniques, MRI, ultrasonography, digital rectal examination (DRE), computed tomography (CT), and cancer protein assay are various clinical diagnostic strategies for PCa detection [120,121]. While all of these methods are powerful and highly successful in detecting of PCa, most of these approaches still complain about lack of precision, sensitivity and specificity for clinical purposes [120,122]. Evidence strongly indicate that the tracking of PCa at the first development stage can help maximize the effectiveness for medical approaches and improve cancer survival from 10% to 90% [120]. Nano-medicine can represent new interventions for the early detection of cancer based on PCa biomarkers, and resultantly, many attempts have been undertaken to develop novel nanotechnology-based diagnostics for early detection of cancers [71,123,124,125]. In biochemistry, a biomarker is considered to be an agent that helps to detect and isolate a specific bio-molecule indicating a specific condition of the a disease [126]. For instance, a larger than a normal level of PSA in the blood serum can be a symptom of prostate cancer [127]. In recent years various PCa biomarkers have been identified. Prostate-specific antigen, also known as human kallikerin 3 (hk3), human kallikerin 2 (hk2), prostate cancer gene 3 (PCA3), prostate stem cell antigen (PSCA), and so on, are the biomarkers that can be used for diagnostic testing and PCa progression tracking [128,129,130]. Such biomarkers may be contained inside the human body’s blood or plasma, urine, semen, and tissues. The most frequently used biomarker for the detection of prostate cancer (PCa) is the PSA or prostate specific antigen. Excessive concentrations of this biomarker (upwards of 4 ng/mL) in blood serum can be a sign of cancer. PSA is being used as the national currency for the initial diagnosis of the PCa since confirmation by the FDA 25 years ago [71,131,132,133]. A graphical representation of the various biomarkers and nano-methodologies for the early detection of PCa is shown in Figure 5.

### 3.1. Nanomaterials for Prostate Cancer Diagnosis and Biosensing

Nanotechnology research brings last-generation strategies for the detection of prostate cancer biomarkers that can fundamentally change the precise management of prostate cancer [135]. The key reason for using structures or materials of nanometer size is to exploit the particular physical properties (including magnetic, optical, structural and electronic properties) that are plainly obvious in within nanometric size [136]. Nanobiosensors only require the interaction of a few bio-target molecules of similar size to create a quick input signals [137]. Due mainly to their extremely sensitive analytical detection features, clinical relevance and accessibility, the development of nanotechnology-based strategies for prostate cancer screening is highly encouraging. Nanotechnology approaches that basically act as general nucleic acid, protein or metabolite biomarker sensors offer remarkable diagnosis performance without any advanced specimen processing methods [138]. Nowadays, many nanoplatforms (such as magnetic nanoparticles, quantum dots, graphene, integrated diagnostics, wearable and implants) have been constructed as a PCa nanosensors (Table 1) [71].

### 3.2. Magnetic Nanoparticles

**Figure 6:**
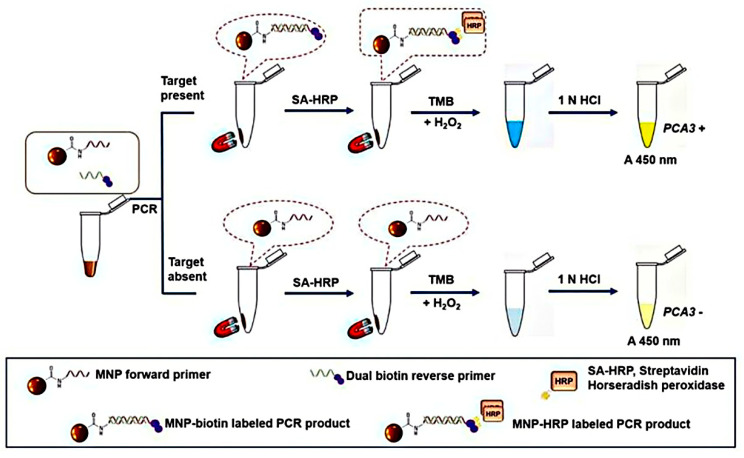
A graphical representation of the MNP-combined oligonucleotide for PCA3 detection in urinary sediments. Reproduced from [94] with permission from Leibniz Research Centre for Working Environment and Human Factors, 2020.

Magnetic nanoparticles (MNPs) were widely utilized due to their unique properties such as magnetic susceptibility, physical characteristics, stability, biocompatibility, ease of mechanism and many more important outcomes [148,149]. MNPs are used to isolate and purify some molecular compounds, like proteins or nucleic acids, before diagnosis [150,151]. This development was indicated for the detection of various biomarkers of prostate cancer proteins in the urine and bloodstream [135,152]. Yamkamon et al. developed Fe_3_O_4_ magnetic nanoparticles-combined with streptavidin-horseradish peroxidase based on PCR method for detecting of urinary PCA3 (a gene specific to prostate cancer). This technique was able to detect PCA3 at femto-gram concentration which was around 1000-fold more effective than traditional RT-PCR. In real sample analysis, PCa patients’ PCA3 expression measured by the prepared nano-platform was greatly higher than that of patients with benign prostatic hyperplasia (BPH) and healthy controls (Figure 6) [94].

There are also multiplex diagnostics to measure the various protein and autoantibody biomarkers from human serum for detection of prostate cancer (PCa). Xu et al. used a four-panel magneto-nano-sensor (MNS) for detection of free PSA/total PSA ratio from human serum. This nano-sensor with array architecture has shown promising potential to separate patients without cancer from those have prostate cancer with high specificity and sensitivity [153].

### 3.3. Gold Nanoparticles

**Figure 7:**
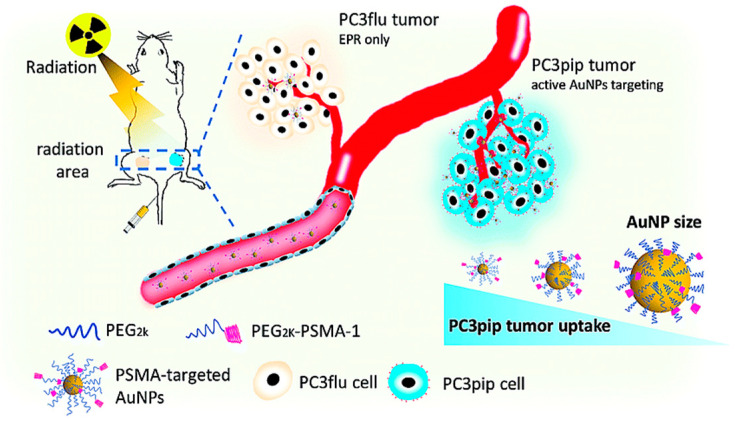
Scheme illustration of targeted prostate cancer radiotherapy using PSMA-targeted AuNPs of various sizes. Reproduced from [158] with permission from Royal Society of Chemistry, 2019.

Gold nanoparticles (AuNPs) exhibited excellent flexibility in medical application, including diagnostic imaging, drug delivery, radiation and photo-therapy [154,155]. Innovations in nano-chemistry and surface chemistry have promoted the creation of AuNPs as nano-biosensors [156]. AuNPs coated with certain hydrophilic polymers exhibit excellent in vivo circulation and high tumor aggregation through the improved permeability and retention effect (EPR) [157]. Lue et al. conjugated PSMA-1 (PCa targeting antigen) to AuNPs for X-ray radiotherapy improvement and observed that the targeting ligand improved gold absorption by PSMA-expressing PC3 pip cells compared to PC3flu cells that lack PSMA receptors (Figure 7) [158].

### 3.4. Quantum Dots

**Figure 8:**
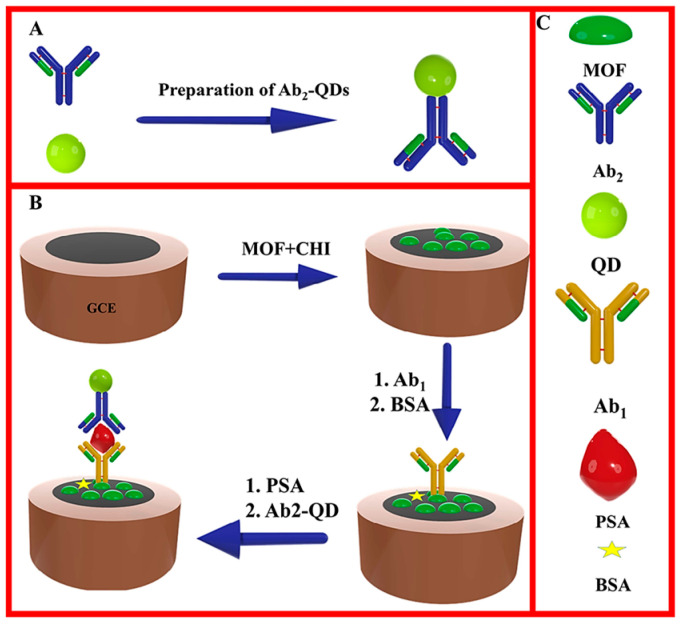
Enzyme-free immunosensor platform based on the Fe_3_O_4_@ TMU-10 MOF magnetic system and nickel-cadmium quantum dots for detection of PSA. Reproduced from [165] with permission from Elsevier, 2020.

Quantum dots (QDs) are semiconducting structures of a nanometer scale with better fluorescence emissions than traditional organic fluorophores due to the quantum confining effect of electron energy bands [159,160]. QDs have ultra-high porous structure, wide surface area, lower electrochemical behavior (higher analytical signal), flexible structure, high electrical and chemical function, etc. [161,162]. The design of developed electrochemical biosensors can be using QDs with such special characteristics [163,164]. Ehzari et al. documented an enzyme-free sandwich immuno-sensor (magnetic structure Fe_3_O_4_@TMU-10 and nickel-cadmium quantum dots) for the PSA biomarker detection. The second antibody, as an electro-active non-enzymatic probe, is cross-linked to a nickel-cadmium quantum dot. The designed immuno-sensor showed a consistent range between 1 pg/mL and 100,000 pg/mL and the 0.45 pg/mL detection limit with appropriate repeatability, specificity, and reliability (Figure 8) [165].

### 3.5. Carbon Nanotubes

**Figure 9:**
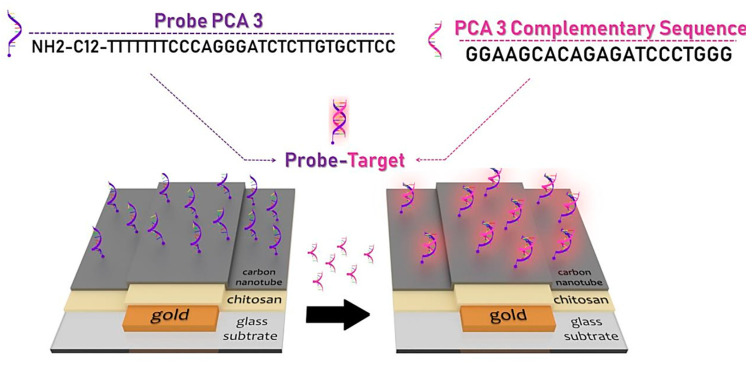
Nano-platform biosensor manufacturing scheme based on carbon nanotubes (MWCNT) for detection of prostate cancer antigen 3 (PCA3). Reproduced from [169] with permission from American Chemical Society, 2019.

Carbon nanotubes (CNTs) are cylindrical, hollow molecules composed of a hexagonal structure of linked carbons, single or multiple walls and a nanometer diameter [166]. CNTs have proved itself as a new styles of superconductors nanoparticles and were used in serum samples and human tissue for electrochemical detection of PSA biomarkers [167,168]. The prostate cancer antigen 3 (PCA3) was found to be much more accurate as a potential biomarker for prostate cancer. Soares et al. reported the first impedance and electrochemically-based nanosensors which are able to detect PCA3 as low as 0.128 nmol/L. The nanosensors were made with a PCA3-complementary single-stranded DNA (ssDNA) probe, immobilized on chitosan (CHT) and carbon nanotubes (MWCNT) layer-by-layer (LbL) film (Figure 9) [169].

### 3.6. Graphene

**Figure 10:**
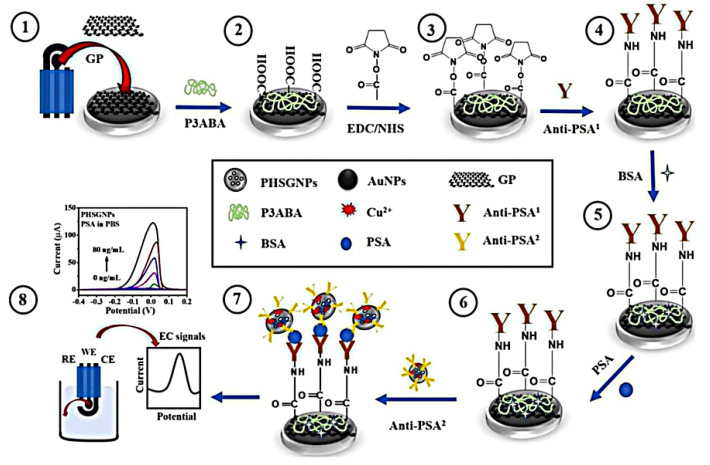
Schematic illustration of biosensing platform (core-shell hollowed-porous-gold-silver nanoparticles (PHSGNPs) and graphene-poly (3-aminobenzoic acid) (GP-P3ABA)) for PSA detection. Reproduced from [175] with permission from Elsevier, 2019.

Graphene plays a significant role in the field of biosensors with extraordinary electrochemical, electrical, magnetic and optical properties [170]. Graphene features, such as functionalization, high flexibility and optical transmittance, have made possible the recent rise of graphene application in nanosensors [171,172]. Numerous studies indicated the possibility for sensitive detection of PCa biomarkers with graphene structures [173,174]. For these reasons, the susceptibility of graphene to PSA biomarkers may lead to earlier diagnosis and hence to a better PCa prognosis. Pothipor et al. developed a graphene based modified electrode for signal amplification of PSA. Their electrode composed of core-shell hollowed-porous-gold-silver nanoparticles (PHSGNPs) and graphene-poly (3-aminobenzoic acid) (GP-P3ABA) (Figure 10). Based on their tests, the sensing efficiency is improved by 120 folds over AuNP labeling and the detection limit (LOD) in human serum hits 0.13 pg/mL or four orders of magnitude better than the clinically appropriate level [175].

Lab-on-a-chip platforms constitute a future generation of PCa biosensors by the development of novel nanomaterials, and nanotechnology has allowed for the design solution-based lab-in-a-drop or lab-on-a-chip systems for miniaturized devices [176,177]. These systems reduce and combine various aspects of the biomarker assessment (such as patient sample collection, target copy amplification and target tracking) into a unique platform that lead to minimizing patient sample requirements and sample-to-outcome response times [178]. Lab-in-a-drop systems are alternate solution nanodiagnostic devices that miniaturize the entire biomarker detection system within a single droplet of fluid [179]. The biggest benefit of lab-in-a-drop systems is the elimination of the need for expensive and skilled precision engineering for detection [180,181].

**Figure 11:**
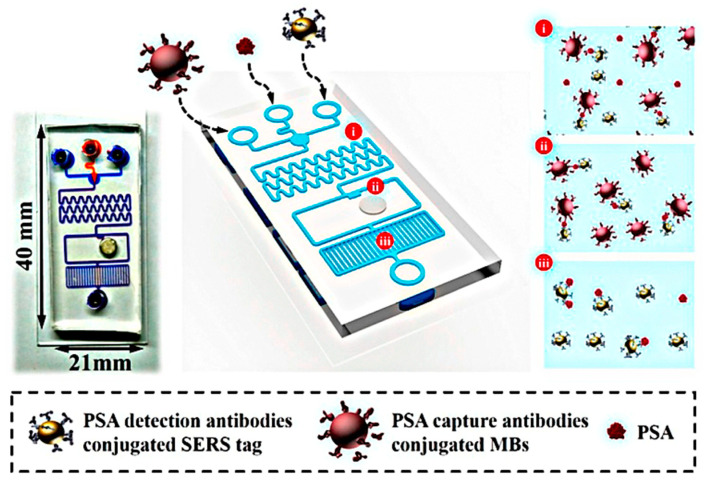
Schematic illustration of the pump-free microfluidic chip for the detection of PSA biomarkers. Reproduced from [182] with permission from American Chemical Society, 2019.

Specific integrated biochips have been designed for the entire sample-to-targeted gene analysis of prostate cancer in urine and serum samples [177]. Gao et al. reported an immunoassay sensor based on surface-enhanced Raman scattering (SERS) with microfluidic technique to quickly detect PSA [182]. The immunoassay platform “sandwich,” consisting of SERS nano-tags, magnetic beads and PSA biomarkers, on a pump-free microfluidic biosensor (Figure 11). Their results revealed strong linear response in the 0.01–100 ng/mL range. Using this chip, the PSA detection limit is estimated to be below 0.01 ng/mL. This PSA biomarker detection level in human serum can be achieved in 5 min without manual incubation or hard dialysis machine [182]. In another study, Feng et al. documented an integrated PSA immunodetection microfluidic chip using a giant magnetoimpedance (GMI) sensor with a detection limit as small as 0.1 ng/mL and operates in the concentration range of 0.1–20 ng/mL [183].

Simultaneous identification of multiple biomarkers plays a significant role in accurate and consistent cancer diagnosis. In a study, the Gao group also developed microfluidic devices based on surface-enhanced Raman scattering (SERS) for the simultaneous detection of total PSA (t-PSA) and free PSA (f-PSA) biomarkers [120]. Their data showed very good linear response from 0.05 to 100 ng/mL for both PSA markers. The detection limits for both the t-PSA and f-PSA were estimated to be below 0.1 ng/mL [120].

## 4. Conclusions and Perspectives

The application of nanomaterials in the field of biomedicines has had a great impact on the delivery of anti-neoplastics. Efficient strategies regarding active targeting are either improved or under clinical evaluation. Farokhzad and Langer along with other researchers have proposed the development of carriers synthesized from biocompatible aptamer polymers to target the PSMA. Besides this study, many similar studies comprising a translation of bioconjugates into clinical practice have resulted in targeted polymeric NPs in targeting PCa. A single biomarker is rarely effective to reach the diagnostic sensitivity and specificity required to allow for accurate stratification of the risk of prostate cancer. Experimental studies over the next years will likely concentrate on clinical assessment and integration of different combinations of next-generation prostate cancer biomarkers. Therefore, the special advantages of nanomaterials could be utilized at a reduced cost for heightened susceptibility, precision, efficiency, and automation. With the many groundbreaking advances in nano-diagnostic methods, the cancer nanotechnology’s potential for improving the treatment of prostate cancer is highly promising. In the near future, researchers must properly evaluate their methodologies in relevant patient cohorts, establish clinically relevant detection limits, and fully evaluate clinical performance parameters to translate these nanotechnologies into clinical usage.

Among all the nanosensors discussed in this review, ultrasensitive analyses of prostate biomarkers can be electrochemical and mass cantilever-based biosensors that present detection limits down to pg/mL. Micro cantilever arrays might provide some level of multimode assessment but an array of up to 10 different biomarkers is most likely sufficient for the identification of multiple cancers, including prostate cancer. Indeed, the most accurate and powerful biosensors for prostate cancer can be lab on a chip devices. Of course, the biosensor interfaces should be accessible to laboratories around the world in the pattern of a compact system to become an alternative to the widely used ELISA method, and their consistency should be measured directly in human serum samples. A high percentage of biosensing devices is still only used in the laboratories, and for academic purposes, there is a need for genuinely reliable devices with high accuracy, good storage stability, and high performance for accurate prostate diagnosis.

## Figures and Tables

**Table 1:** Nano-methodology approaches used in the biosensing of prostate cancer biomarkers.

Nanomethodology	Biomarker	Detection Medium	Feature	Ref.
Magnetic nanoparticle	PSA	Human plasma	Appropriate linear range between 0.001 and 1 μg/L (via SWV method) with a 0.001 μg/L LLOQ.	[139]
Gold nanoparticles	PSA	Serums of healthy and prostate patients	Linear range 0~0.8 ng/mL for PSA measurement with a detection maximum of 0.02 ng/mL.	[140]
Silicon nanowires	miRNA 183 and 484	Plasma	Target nucleic acid molecules can be detected with a high sensitivity of 3.3 × 10^−16^ M.	[141]
Quantum dots	f-PSA and cPSA	Two human serum	At the same time, detect f-PSA and c-PSA with detection limits of 0.009 ng/mL, in a quick assay time of 60 min.	[142]
Carbon nanotubes	miR-21	Human serum	Strong linear relation with miR-21 target concentration (0.01 fmol/L to 1 μmol/L) and low experimental detection limit of 0.01 fmol/L.	[143]
Graphene	PSA	Blood	The detection limit for total and free PSA antigen was about 0.2 and 0.07 ng/mL, respectively.	[144]
Surface-enhanced Raman scattering (SERS) nanoparticles	PSA	Blood serum	Technique can substantially distinguish between low-risk and high-risk PCa with 92.3% accuracy, 89.5% sensitivity and 95% specificity.	[145]
Micro-cantilever or Piezoelectric material	PSA	HP and HSA	Offer a strong platform for DNA-protein, protein-protein binding, and DNA hybridization interactions with high-throughput label-free analyzes.	[146]
Lab-on-a-chip systems	PSA, PSMA and PF-4	Serum	Detection limits for the 3 proteins in undiluted calf serum was 300–500 fg/mL.	[147]

Cancer Prostate (PCa), Free and complexed prostate-specific antigen (f-PSA and c-PSA), MicroRNAs (miRNAs), human serum albumin (HSA), human plasminogen (HP), prostate specific antigen (PSA), prostate specific membrane antigen (PSMA) and platelet factor-4 (PF-4).
